# Crystal structure of *tert*-butyl 3,6-di­iodo­carbazole-9-carboxyl­ate

**DOI:** 10.1107/S205698902300230X

**Published:** 2023-03-15

**Authors:** Elizabeth N. Sielaff, Richard J. Staples, Shannon M. Biros

**Affiliations:** a Department of Chemistry, Grand Valley State University, Allendale, MI 49401, USA; b Center for Crystallographic Research, Department of Chemistry and Chemical Biology, Michigan State University, East Lansing, MI 48824, USA; Vienna University of Technology, Austria

**Keywords:** crystal structure, carbazole, halogen–halogen inter­action, π–π inter­action

## Abstract

The mol­ecular structure of *tert*-butyl 3,6-di­iodo­carbazole-9-carboxyl­ate, C_17_H_15_I_2_NO_2_, features a nearly planar 13-membered carbazole ring with C—I bond lengths of 2.092 (4) and 2.104 (4) Å. The carbamate group has key bond lengths of 1.404 (6) Å (N—C), 1.330 (5) Å (O—C), and 1.201 (6) Å (C=O). The crystal contains inter­molecular π–π inter­actions, as well as both type I and type II inter­molecular I⋯I inter­actions.

## Abstract 2

The crystal structure of *tert*-butyl 3,6-di­iodo­carbazole-9-carboxyl­ate features inter­molecular π–π inter­actions, as well as both type I and type II inter­molecular I⋯I inter­actions.

## 1. Chemical context

Derivatives of the carbazole ring system have been used in a wide variety of applications ranging from organic light-emitting diodes (Uoyama *et al.*, 2012 ▸) to cell membrane targeting fluorescent probes (Wnag *et al.*, 2023 ▸) to compounds that are able to influence the supra­molecular structure of G-rich DNA sequences (Debnath *et al.*, 2016 ▸). Our group’s inter­est in this mol­ecular entity was inspired by the work of de Bettencourt-Dias and co-workers who have used carbazole derivatives as antennas to sensitize the luminescence of lanthanide metals (Monteiro *et al.*, 2017 ▸, 2018 ▸, 2020 ▸, 2022 ▸). Our group was working to derivatize carbazole for use in related lanthanide luminescence applications when compound **I**, a synthetic inter­mediate in our work, serendipitously crystallized in an NMR tube. 
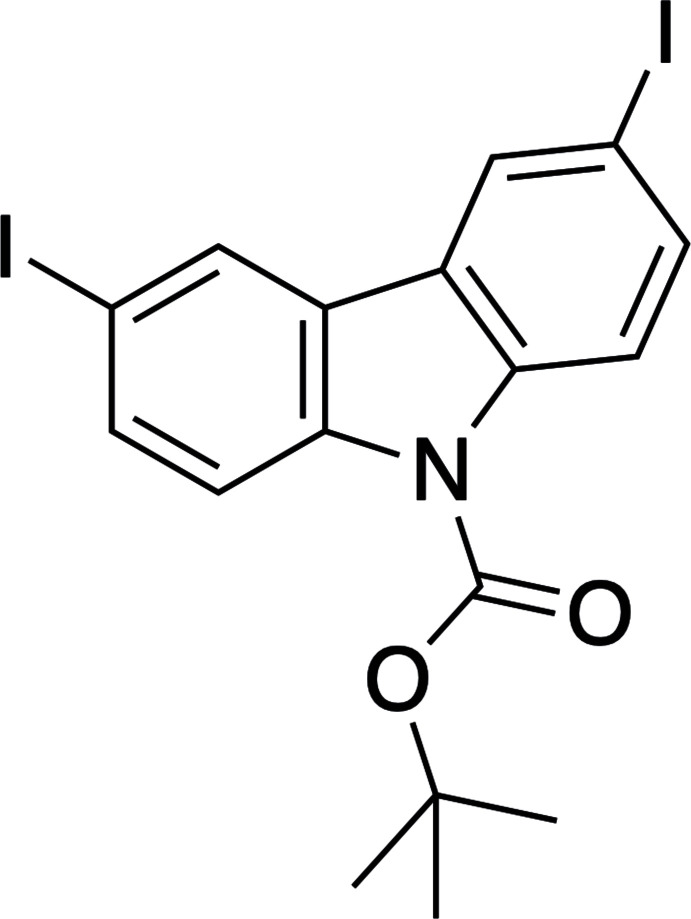


## 2. Structural commentary

**Figure 1:**
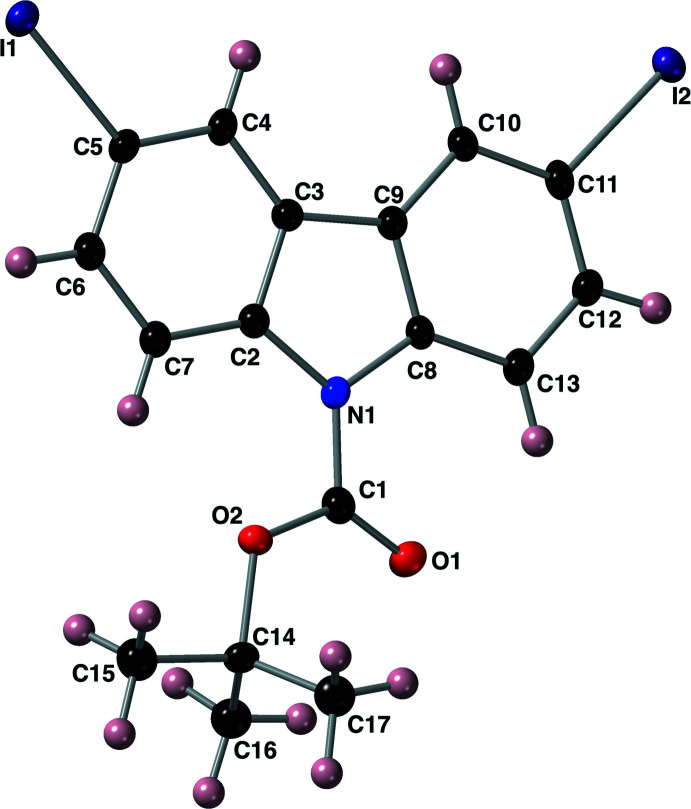
The mol­ecular structure of compound **I**, with the atom-labelling scheme. Displacement ellipsoids are drawn at the 50% probability level using standard CPK colors (I = purple).

The mol­ecular structure of compound **I** is shown in Fig. 1 ▸ along with the atom-numbering scheme. The structure of this substituted carbazole has C—I bond lengths of 2.092 (4) and 2.104 (4) Å. The carbamate group has bond lengths of 1.404 (6) Å for N1—C1, 1.330 (5) Å for O2—C1, and 1.201 (6) Å for the carbonyl C1=O1. The N1—C1—O2 bond angle is 110.3 (4)°, and these atoms are roughly coplanar with the atoms of the aromatic system with a C8—N1—C1—O2 torsion angle of −7.3 (7)°. The 13-membered aromatic carbazole ring approaches planarity with an r.m.s. deviation of 0.007 Å where the atom C8 deviates the most from the calculated least-squares plane by 0.019 (4) Å.

## 3. Supra­molecular features

**Figure 2:**
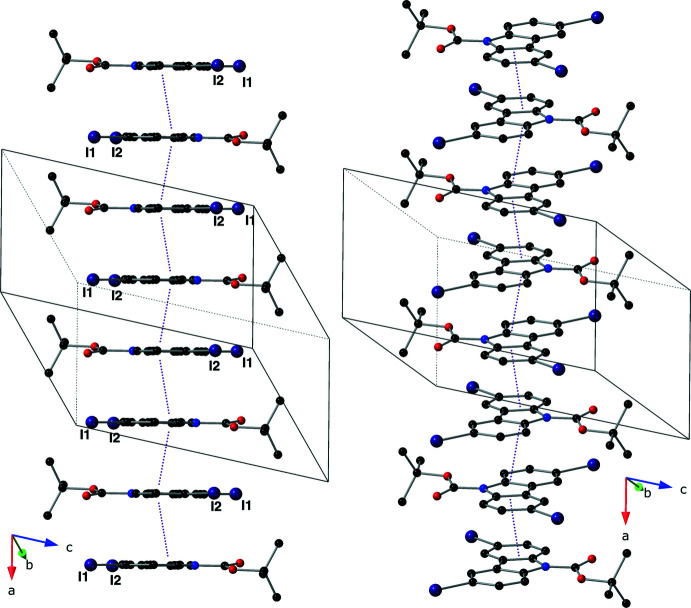
A depiction of the supra­molecular pillars using a ball-and-stick model with standard CPK colors (I = purple). The left portion of the figure shows the pillars with a view that is aligned with the plane of the aromatic carbazole system, the right portion of the figure shows the same mol­ecules tilted slightly along the *b* axis. The π–π inter­actions described in the text are depicted with purple dotted lines and the unit cell is drawn with a solid black line.

**Figure 3:**
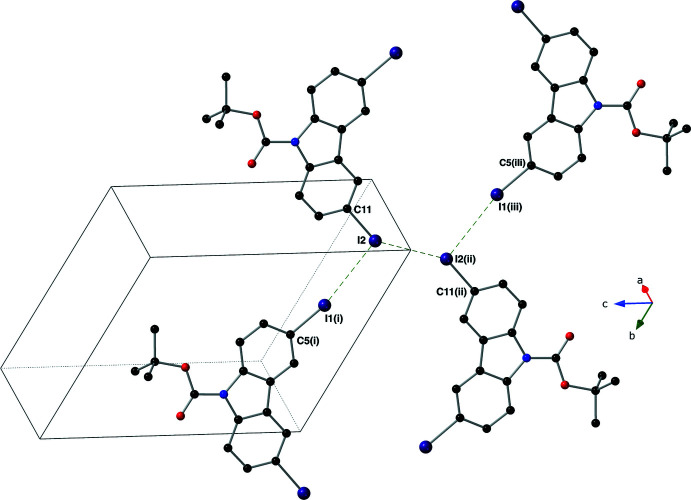
A figure showing the halogen–halogen inter­actions (depicted as green, dashed lines) present in the crystal of compound **I** using a ball-and-stick model with standard CPK colors (I = purple). The unit cell is drawn with a solid black line. [Symmetry codes: (i) −*x* + 1, −*y* + 1, −*z* + 2, (ii) −*x* + 2, −*y*, −*z* + 2, (iii) *x* + 1, *y* − 1, *z*.]

In the crystal, mol­ecules of the title compound form pillars *via* inter­molecular π–π inter­actions that propagate parallel to the *a* axis; the centroid of the five-membered ring (N1/C2/C3/C9/C8) is denoted as *Cg*. These inter­actions have *Cg*⋯*Cg* distances of 3.484 (3) and 3.589 (3) Å with slippages of 1.028 and 1.376 Å and angles of 0.00 (3)° (Fig. 2 ▸; symmetry codes: −*x* + 1, −*y* + 1, −*z* + 1 and −*x* + 2, −*y* + 1, −*z* + 1). The supra­molecular pillars are held together *via* both type I and type II inter­molecular I⋯I inter­actions (Pedireddi *et al.*, 1994 ▸; Figs. 3 ▸ and 4 ▸). The type I halogen–halogen inter­action has a *trans* arrangement and exists between atoms C11—I2⋯I2(−*x* + 2, −*y*, –*z* + 2) with an angle of 147.68 (13)° and an I⋯I distance of 3.6630 (5) Å. The type II inter­action is found between atoms C11—I2⋯I1(*x* + 1, *y* − 1, *z*) with an I⋯I distance of 3.8332 (5) Å and an angle of 46.69 (13)°.

## 4. Database survey

A search of the Cambridge Structure Database (CSD version 5.43 with updates through June 2022; Groom *et al.*, 2016 ▸) for structures containing the carbazole ring system substituted with any halogen atom at the C5 and C11 positions (as numbered in Fig. 1 ▸) returned 101 hits. The structures CEYXAI (Malecki, 2018 ▸) and FUMLIK (Radula-Janik *et al.*, 2015 ▸) are closely related to that of compound **I** with iodine atoms at the C5 and C11 positions, but where the nitro­gen atom has been alkyl­ated with either a butyl or benzyl group. Structure ECUNUM bears bromine atoms at the C5 and C11 positions with a phenyl­carbamate group on the nitro­gen atom (Duan *et al.*, 2006 ▸). A derivative of compound **I** that bears two iodine atoms in the same positions and a hydrogen atom bonded to the nitro­gen atom has been solved as structure YAYDUZ (Xie *et al.*, 2012 ▸). Lastly, the di-iodo carbazole has been used as a ligand in a copper(I) complex as demonstrated by Kim and co-workers (ZASYUQ; Kim *et al.*, 2017 ▸).

## 5. Synthesis and crystallization

The title compound was prepared according to the procedure published by Lee and co-workers (Moon *et al.*, 2007 ▸). The compound was dissolved in CDCl_3_ and the crystals studied here grew as the solvent slowly evaporated from the NMR tube.

## 6. Refinement

Crystal data, data collection and structure refinement details are summarized in Table 1 ▸. All hydrogen atoms bonded to carbon atoms were placed in calculated positions and refined as riding: C—H = 0.95–1.00 Å with *U*_iso_(H) = 1.2*U*_eq_(C) for aromatic hydrogen atoms and *U*_iso_(H) = 1.5*U*_eq_(C) for the hydrogen atoms of the methyl group.

## Supplementary Material

Crystal structure: contains datablock(s) I. DOI: 10.1107/S205698902300230X/wm5676sup1.cif

Structure factors: contains datablock(s) I. DOI: 10.1107/S205698902300230X/wm5676Isup3.hkl

Supporting information file. DOI: 10.1107/S205698902300230X/wm5676Isup3.cml

CCDC reference: 2247288

Additional supporting information:  crystallographic information; 3D view; checkCIF report

## Figures and Tables

**Figure 4:**
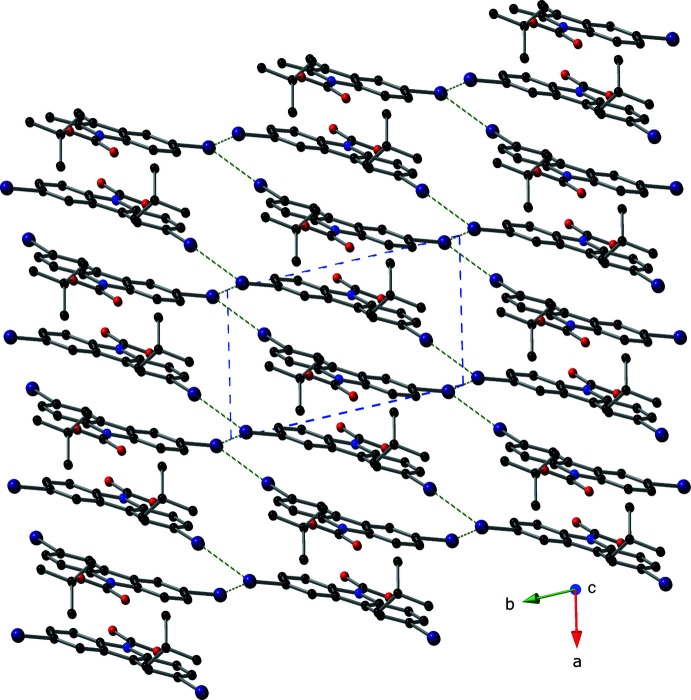
A view down the *c* axis showing the supra­molecular pillars found in the crystal of compound **I** as well as the I—I inter­actions (depicted as green, dashed lines) that hold them together using a ball-and-stick model with standard CPK colors (I = purple). The unit cell is drawn with a blue, dashed line and the π—π inter­actions are not shown for clarity.

**Table 1:** Experimental details

Crystal data	
Chemical formula	C_17_H_15_I_2_NO_2_
*M*_r_	519.10
Crystal system, space group	Triclinic, *P* 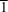
Temperature (K)	100
*a*, *b*, *c* (Å)	6.9611 (3), 11.9737 (5), 12.0697 (4)
α, β, γ (°)	65.618 (4), 78.588 (3), 74.826 (4)
*V* (Å^3^)	879.71 (7)
*Z*	2
Radiation type	Cu *K*α
μ (mm^−1^)	28.13
Crystal size (mm)	0.11 × 0.06 × 0.02

Data collection	
Diffractometer	XtaLAB Synergy, Dualflex, HyPix
Absorption correction	Multi-scan (*CrysAlis PRO*; Oxford Diffraction, 2006 ▸)
*T*_min_, *T*_max_	0.756, 1.000
No. of measured, independent and observed [*I* > 2σ(*I*)] reflections	10800, 3717, 3209
*R*_int_	0.038
(sin θ/λ)_max_ (Å^−1^)	0.639

Refinement	
*R*[*F* ^2^ > 2σ(*F* ^2^)], *wR*(*F* ^2^), *S*	0.033, 0.090, 1.08
No. of reflections	3717
No. of parameters	202
H-atom treatment	H-atom parameters constrained
Δρ_max_, Δρ_min_ (e Å^−3^)	0.74, −1.17

Computer programs: *CrysAlis PRO* (Oxford Diffraction, 2006 ▸), *SHELXT* (Sheldrick, 2015*a* ▸), *SHELXL* (Sheldrick, 2015*b* ▸), *CrystalMaker* (Palmer, 2007 ▸), *OLEX2* (Dolomanov *et al.*, 2009 ▸; Bourhis *et al.*, 2015 ▸).

## supplementary crystallographic information

### Crystal data

**Table:**

C_17_H_15_I_2_NO_2_	*Z* = 2
*M**_r_* = 519.10	*F*(000) = 492
Triclinic, *P*1	*D*_x_ = 1.960 Mg m^−^^3^
*a* = 6.9611 (3) Å	Cu *K*α radiation, λ = 1.54184 Å
*b* = 11.9737 (5) Å	Cell parameters from 6514 reflections
*c* = 12.0697 (4) Å	θ = 4.1–79.3°
α = 65.618 (4)°	µ = 28.13 mm^−^^1^
β = 78.588 (3)°	*T* = 100 K
γ = 74.826 (4)°	Irregular, colourless
*V* = 879.71 (7) Å^3^	0.11 × 0.06 × 0.02 mm

### Data collection

**Table:**

XtaLAB Synergy, Dualflex, HyPix diffractometer	3717 independent reflections
Radiation source: micro-focus sealed X-ray tube, PhotonJet (Cu) X-ray Source	3209 reflections with *I* > 2σ(*I*)
Mirror monochromator	*R*_int_ = 0.038
Detector resolution: 10.0000 pixels mm^-1^	θ_max_ = 80.0°, θ_min_ = 4.0°
ω scans	*h* = −8→8
Absorption correction: multi-scan (CrysAlisPro; Oxford Diffraction, 2006)	*k* = −15→15
*T*_min_ = 0.756, *T*_max_ = 1.000	*l* = −15→12
10800 measured reflections	

### Refinement

**Table:**

Refinement on *F*^2^	Primary atom site location: dual
Least-squares matrix: full	Hydrogen site location: inferred from neighbouring sites
*R*[*F*^2^ > 2σ(*F*^2^)] = 0.033	H-atom parameters constrained
*wR*(*F*^2^) = 0.090	*w* = 1/[σ^2^(*F*_o_^2^) + (0.0414*P*)^2^ + 2.1415*P*] where *P* = (*F*_o_^2^ + 2*F*_c_^2^)/3
*S* = 1.08	(Δ/σ)_max_ = 0.001
3717 reflections	Δρ_max_ = 0.74 e Å^−^^3^
202 parameters	Δρ_min_ = −1.17 e Å^−^^3^
0 restraints	

### Special details

**Table:**

Geometry. All esds (except the esd in the dihedral angle between two l.s. planes) are estimated using the full covariance matrix. The cell esds are taken into account individually in the estimation of esds in distances, angles and torsion angles; correlations between esds in cell parameters are only used when they are defined by crystal symmetry. An approximate (isotropic) treatment of cell esds is used for estimating esds involving l.s. planes.

### Fractional atomic coordinates and isotropic or equivalent isotropic displacement parameters (Å^2^)

**Table:**

	*x*	*y*	*z*	*U*_iso_*/*U*_eq_	
I1	0.35523 (5)	0.84654 (3)	0.68070 (3)	0.02553 (10)	
I2	1.02078 (5)	0.06482 (2)	0.83259 (2)	0.02519 (10)	
O1	0.9081 (6)	0.4713 (3)	0.2038 (3)	0.0308 (8)	
O2	0.6990 (5)	0.6592 (3)	0.1740 (3)	0.0229 (7)	
N1	0.7721 (6)	0.5314 (3)	0.3644 (3)	0.0176 (7)	
C1	0.8021 (7)	0.5484 (4)	0.2405 (4)	0.0216 (9)	
C2	0.6704 (7)	0.6182 (4)	0.4183 (4)	0.0177 (8)	
C3	0.6784 (7)	0.5579 (4)	0.5462 (4)	0.0178 (8)	
C4	0.5909 (7)	0.6212 (4)	0.6236 (4)	0.0199 (9)	
H4	0.596672	0.580826	0.709538	0.024*	
C5	0.4954 (7)	0.7448 (4)	0.5712 (4)	0.0189 (8)	
C6	0.4877 (7)	0.8063 (4)	0.4439 (4)	0.0206 (9)	
H6	0.423151	0.891758	0.410340	0.025*	
C7	0.5745 (7)	0.7422 (4)	0.3669 (4)	0.0194 (8)	
H7	0.567988	0.782539	0.281049	0.023*	
C8	0.8459 (6)	0.4167 (4)	0.4582 (4)	0.0169 (8)	
C9	0.7881 (6)	0.4311 (4)	0.5706 (4)	0.0176 (8)	
C10	0.8392 (7)	0.3303 (4)	0.6804 (4)	0.0190 (8)	
H10	0.802819	0.338279	0.757291	0.023*	
C11	0.9449 (7)	0.2191 (4)	0.6707 (4)	0.0201 (9)	
C12	0.9994 (7)	0.2046 (4)	0.5597 (4)	0.0207 (9)	
H12	1.070569	0.125606	0.558551	0.025*	
C13	0.9521 (7)	0.3030 (4)	0.4505 (4)	0.0204 (9)	
H13	0.989829	0.293776	0.374211	0.024*	
C14	0.7063 (8)	0.6986 (4)	0.0389 (4)	0.0244 (10)	
C15	0.5773 (9)	0.8287 (5)	0.0027 (5)	0.0332 (12)	
H15A	0.442886	0.824282	0.045892	0.050*	
H15B	0.568722	0.864481	−0.085716	0.050*	
H15C	0.636703	0.881733	0.024436	0.050*	
C16	0.9203 (9)	0.7015 (5)	−0.0200 (5)	0.0336 (12)	
H16A	0.980140	0.748546	0.009296	0.050*	
H16B	0.920443	0.742127	−0.109047	0.050*	
H16C	0.998346	0.615734	0.001846	0.050*	
C17	0.6131 (10)	0.6110 (5)	0.0144 (5)	0.0351 (12)	
H17A	0.688431	0.525099	0.050166	0.053*	
H17B	0.616737	0.635693	−0.074038	0.053*	
H17C	0.474017	0.615706	0.051213	0.053*	

### Atomic displacement parameters (Å^2^)

**Table:**

	*U*^11^	*U*^22^	*U*^33^	*U*^12^	*U*^13^	*U*^23^
I1	0.03315 (18)	0.01775 (15)	0.02357 (16)	0.00301 (11)	−0.00265 (12)	−0.01080 (12)
I2	0.03258 (18)	0.01525 (15)	0.01960 (16)	0.00277 (11)	−0.00287 (11)	−0.00313 (11)
O1	0.044 (2)	0.0203 (16)	0.0229 (16)	0.0062 (15)	−0.0023 (15)	−0.0109 (14)
O2	0.0316 (18)	0.0177 (15)	0.0150 (14)	0.0022 (13)	−0.0047 (12)	−0.0053 (12)
N1	0.0192 (18)	0.0143 (16)	0.0183 (17)	−0.0010 (14)	−0.0004 (14)	−0.0071 (14)
C1	0.021 (2)	0.017 (2)	0.022 (2)	0.0003 (17)	−0.0045 (17)	−0.0055 (18)
C2	0.017 (2)	0.017 (2)	0.019 (2)	−0.0053 (16)	−0.0010 (16)	−0.0066 (16)
C3	0.017 (2)	0.016 (2)	0.018 (2)	0.0000 (16)	−0.0008 (16)	−0.0068 (16)
C4	0.021 (2)	0.016 (2)	0.024 (2)	−0.0005 (16)	−0.0029 (17)	−0.0093 (17)
C5	0.017 (2)	0.016 (2)	0.022 (2)	0.0012 (16)	0.0015 (16)	−0.0105 (17)
C6	0.020 (2)	0.016 (2)	0.023 (2)	−0.0018 (16)	−0.0012 (17)	−0.0066 (17)
C7	0.019 (2)	0.016 (2)	0.023 (2)	−0.0011 (16)	−0.0043 (16)	−0.0074 (17)
C8	0.0157 (19)	0.0137 (19)	0.019 (2)	−0.0014 (15)	−0.0013 (15)	−0.0054 (16)
C9	0.0150 (19)	0.016 (2)	0.021 (2)	−0.0013 (15)	0.0015 (15)	−0.0076 (16)
C10	0.018 (2)	0.0145 (19)	0.020 (2)	−0.0001 (16)	−0.0010 (16)	−0.0048 (17)
C11	0.021 (2)	0.0119 (19)	0.023 (2)	0.0024 (16)	−0.0087 (17)	−0.0025 (16)
C12	0.022 (2)	0.0156 (19)	0.023 (2)	0.0000 (16)	−0.0016 (17)	−0.0087 (17)
C13	0.019 (2)	0.017 (2)	0.025 (2)	−0.0011 (16)	−0.0008 (17)	−0.0094 (17)
C14	0.035 (3)	0.021 (2)	0.017 (2)	−0.0024 (19)	−0.0028 (18)	−0.0082 (18)
C15	0.040 (3)	0.023 (2)	0.029 (3)	0.006 (2)	−0.010 (2)	−0.007 (2)
C16	0.040 (3)	0.027 (3)	0.029 (3)	−0.003 (2)	0.005 (2)	−0.012 (2)
C17	0.052 (4)	0.028 (3)	0.027 (3)	−0.009 (2)	−0.011 (2)	−0.009 (2)

### Geometric parameters (Å, º)

**Table:**

I1—C5	2.092 (4)	C9—C10	1.408 (6)
I2—C11	2.104 (4)	C10—H10	0.9500
O1—C1	1.201 (6)	C10—C11	1.382 (6)
O2—C1	1.330 (5)	C11—C12	1.388 (7)
O2—C14	1.493 (5)	C12—H12	0.9500
N1—C1	1.404 (6)	C12—C13	1.386 (6)
N1—C2	1.413 (6)	C13—H13	0.9500
N1—C8	1.416 (6)	C14—C15	1.512 (7)
C2—C3	1.414 (6)	C14—C16	1.521 (8)
C2—C7	1.385 (6)	C14—C17	1.516 (7)
C3—C4	1.391 (6)	C15—H15A	0.9800
C3—C9	1.446 (6)	C15—H15B	0.9800
C4—H4	0.9500	C15—H15C	0.9800
C4—C5	1.383 (6)	C16—H16A	0.9800
C5—C6	1.409 (6)	C16—H16B	0.9800
C6—H6	0.9500	C16—H16C	0.9800
C6—C7	1.393 (6)	C17—H17A	0.9800
C7—H7	0.9500	C17—H17B	0.9800
C8—C9	1.404 (6)	C17—H17C	0.9800
C8—C13	1.400 (6)		
			
C1—O2—C14	120.3 (4)	C10—C11—I2	118.1 (3)
C1—N1—C2	128.7 (4)	C10—C11—C12	122.9 (4)
C1—N1—C8	122.7 (4)	C12—C11—I2	119.0 (3)
C2—N1—C8	108.5 (3)	C11—C12—H12	119.4
O1—C1—O2	127.0 (4)	C13—C12—C11	121.3 (4)
O1—C1—N1	122.8 (4)	C13—C12—H12	119.4
O2—C1—N1	110.3 (4)	C8—C13—H13	121.6
N1—C2—C3	108.2 (4)	C12—C13—C8	116.7 (4)
C7—C2—N1	131.2 (4)	C12—C13—H13	121.6
C7—C2—C3	120.7 (4)	O2—C14—C15	101.9 (4)
C2—C3—C9	107.3 (4)	O2—C14—C16	110.3 (4)
C4—C3—C2	121.1 (4)	O2—C14—C17	108.4 (4)
C4—C3—C9	131.6 (4)	C15—C14—C16	111.4 (4)
C3—C4—H4	121.2	C15—C14—C17	111.3 (5)
C5—C4—C3	117.7 (4)	C17—C14—C16	113.0 (5)
C5—C4—H4	121.2	C14—C15—H15A	109.5
C4—C5—I1	120.4 (3)	C14—C15—H15B	109.5
C4—C5—C6	121.8 (4)	C14—C15—H15C	109.5
C6—C5—I1	117.8 (3)	H15A—C15—H15B	109.5
C5—C6—H6	119.9	H15A—C15—H15C	109.5
C7—C6—C5	120.3 (4)	H15B—C15—H15C	109.5
C7—C6—H6	119.9	C14—C16—H16A	109.5
C2—C7—C6	118.5 (4)	C14—C16—H16B	109.5
C2—C7—H7	120.8	C14—C16—H16C	109.5
C6—C7—H7	120.8	H16A—C16—H16B	109.5
C9—C8—N1	108.2 (4)	H16A—C16—H16C	109.5
C13—C8—N1	129.7 (4)	H16B—C16—H16C	109.5
C13—C8—C9	122.1 (4)	C14—C17—H17A	109.5
C8—C9—C3	107.9 (4)	C14—C17—H17B	109.5
C8—C9—C10	120.4 (4)	C14—C17—H17C	109.5
C10—C9—C3	131.8 (4)	H17A—C17—H17B	109.5
C9—C10—H10	121.7	H17A—C17—H17C	109.5
C11—C10—C9	116.6 (4)	H17B—C17—H17C	109.5
C11—C10—H10	121.7		
			
I1—C5—C6—C7	−178.5 (3)	C3—C4—C5—C6	−0.7 (7)
I2—C11—C12—C13	179.6 (4)	C3—C9—C10—C11	179.1 (5)
N1—C2—C3—C4	179.7 (4)	C4—C3—C9—C8	−179.1 (5)
N1—C2—C3—C9	0.0 (5)	C4—C3—C9—C10	1.1 (9)
N1—C2—C7—C6	−179.2 (4)	C4—C5—C6—C7	1.2 (7)
N1—C8—C9—C3	−0.8 (5)	C5—C6—C7—C2	−1.1 (7)
N1—C8—C9—C10	179.1 (4)	C7—C2—C3—C4	−0.1 (7)
N1—C8—C13—C12	−178.0 (4)	C7—C2—C3—C9	−179.8 (4)
C1—O2—C14—C15	−177.9 (4)	C8—N1—C1—O1	−7.3 (7)
C1—O2—C14—C16	−59.5 (6)	C8—N1—C1—O2	173.0 (4)
C1—O2—C14—C17	64.7 (6)	C8—N1—C2—C3	−0.5 (5)
C1—N1—C2—C3	179.7 (4)	C8—N1—C2—C7	179.2 (5)
C1—N1—C2—C7	−0.6 (8)	C8—C9—C10—C11	−0.7 (7)
C1—N1—C8—C9	−179.3 (4)	C9—C3—C4—C5	179.7 (5)
C1—N1—C8—C13	−1.6 (7)	C9—C8—C13—C12	−0.5 (7)
C2—N1—C1—O1	172.5 (5)	C9—C10—C11—I2	−179.0 (3)
C2—N1—C1—O2	−7.2 (7)	C9—C10—C11—C12	−0.3 (7)
C2—N1—C8—C9	0.8 (5)	C10—C11—C12—C13	0.9 (8)
C2—N1—C8—C13	178.6 (5)	C11—C12—C13—C8	−0.5 (7)
C2—C3—C4—C5	0.2 (7)	C13—C8—C9—C3	−178.8 (4)
C2—C3—C9—C8	0.5 (5)	C13—C8—C9—C10	1.1 (7)
C2—C3—C9—C10	−179.4 (5)	C14—O2—C1—O1	0.5 (8)
C3—C2—C7—C6	0.6 (7)	C14—O2—C1—N1	−179.8 (4)
C3—C4—C5—I1	178.9 (3)		
